# Mitochondrial Function, Fatty Acid Metabolism, and Body Composition in the Hyperbilirubinemic Gunn Rat

**DOI:** 10.3389/fphar.2021.586715

**Published:** 2021-03-08

**Authors:** Josif Vidimce, Johara Pillay, Nirajan Shrestha, Lan-feng Dong, Jiri Neuzil, Karl-Heinz Wagner, Olivia Jane Holland, Andrew Cameron Bulmer

**Affiliations:** ^1^ School of Medical Science, Griffith University, Gold Coast, QLD, Australia; ^2^ Institute of Biotechnology, Czech Academy of Sciences, Prague, Czechia; ^3^ Department of Nutritional Sciences and Research Platform Active Ageing, University of Vienna, Vienna, Austria

**Keywords:** lipids, respiration, metabolism, mitochondria, Gunn rat, triglycerides, unconjugated bilirubin (UCB), hyperbilirubinemia

## Abstract

**Background:** Circulating bilirubin is associated with reduced adiposity in human and animal studies. A possible explanation is provided by *in vitro* data that demonstrates that bilirubin inhibits mitochondrial function and decreases efficient energy production. However, it remains unclear whether hyperbilirubinemic animals have similar perturbed mitochondrial function and whether this is important for regulation of energy homeostasis.

**Aim:** To investigate the impact of unconjugated hyperbilirubinemia on body composition, and mitochondrial function in hepatic tissue and skeletal muscle.

**Materials and Methods:** 1) Food intake and bodyweight gain of 14-week old hyperbilirubinemic Gunn (*n* = 19) and normobilirubinemic littermate (control; *n* = 19) rats were measured over a 17-day period. 2) Body composition was determined using dual-energy X-ray absorptiometry and by measuring organ and skeletal muscle masses. 3) Mitochondrial function was assessed using high-resolution respirometry of homogenized liver and intact permeabilized extensor digitorum longus and soleus fibers. 4) Liver tissue was flash frozen for later gene (qPCR), protein (Western Blot and citrate synthase activity) and lipid analysis.

**Results:** Female hyperbilirubinemic rats had significantly reduced fat mass (Gunn: 9.94 ± 5.35 vs. Control: 16.6 ± 6.90 g, *p* < 0.05) and hepatic triglyceride concentration (Gunn: 2.39 ± 0.92 vs. Control: 4.65 ± 1.67 mg g^−1^, *p* < 0.01) compared to normobilirubinemic controls. Furthermore, hyperbilirubinemic rats consumed fewer calories daily (*p* < 0.01) and were less energetically efficient (Gunn: 8.09 ± 5.75 vs. Control: 14.9 ± 5.10 g bodyweight kcal^−1^, *p* < 0.05). Hepatic mitochondria of hyperbilirubinemic rats demonstrated increased flux control ratio (FCR) via complex I and II (CI+II) (Gunn: 0.78 ± 0.16 vs. Control: 0.62 ± 0.09, *p* < 0.05). Similarly, exogenous addition of 31.3 or 62.5 μM unconjugated bilirubin to control liver homogenates significantly increased CI+II FCR (*p* < 0.05). Hepatic *PGC-1α* gene expression was significantly increased in hyperbilirubinemic females while *FGF21* and *ACOX1* was significantly greater in male hyperbilirubinemic rats (*p* < 0.05). Finally, hepatic mitochondrial complex IV subunit 1 protein expression was significantly increased in female hyperbilirubinemic rats (*p* < 0.01).

**Conclusions:** This is the first study to comprehensively assess body composition, fat metabolism, and mitochondrial function in hyperbilirubinemic rats. Our findings show that hyperbilirubinemia is associated with reduced fat mass, and increased hepatic mitochondrial biogenesis, specifically in female animals, suggesting a dual role of elevated bilirubin and reduced UGT1A1 function on adiposity and body composition.

## Introduction

Bilirubin is a breakdown product of heme catabolism and it is used clinically to assist in diagnosis of liver dysfunction and blood disorders (Fevery, 2008). A central dogma asserts that unconjugated bilirubin (UCB) is toxic due to its potential to accumulate in specific regions of the brain, and induce oxidative stress, mitochondrial dysfunction, and apoptosis (Rodrigues et al., 2002a; Watchko and Tiribelli, 2013). At physiological pH, UCB has strong intramolecular hydrogen bonding that renders it hydrophobic, consequently, in the circulation UCB is solubilized by strong binding to albumin (Rolf and Stern, 1980; Fevery, 2008). Unbound UCB has low aqueous solubility (<70 nM) (Ostrow et al., 2004) and accumulates within hydrophobic regions of lipid membranes, including the mitochondria (Mustafa and King, 1970; Ostrow et al., 2004).

UCB affects mitochondria by increasing permeability, oxidative damage, and uncoupling of the mitochondrial membrane potential (Ostrow et al., 2004; Watchko and Tiribelli, 2013). Rodrigues et al., (2000) demonstrated that incubation of primary rat neurons with UCB induced mitochondrial swelling and membrane permeability, release of cytochrome c, and apoptosis. Spin-labelling investigations have reported that UCB-induced mitochondrial membrane permeability and lipid peroxidation is related to superficial association of UCB to phospholipid bilayers of the inner mitochondrial membrane (Zucker et al., 1992; Rodrigues et al., 2002b). Together, these findings corroborate the prevailing notion that UCB perturbs membrane dynamics by interacting with lipids (Zucker et al., 1992; Rodrigues et al., 2002b).

Skeletal muscle and liver are the major determinants of basal metabolic rate (BMR) contributing to more than 50% of total BMR in rodents (Kummitha et al., 2014). Under physiological conditions, dietary substrates are oxidized primarily by the mitochondrial electron transport chain (ETC) to produce energy in the form of ATP. However, a fraction of this energy produces heat instead of ATP due to a benign rate of proton leak across the inner mitochondrial membrane, known as (unregulated) mitochondrial uncoupling (Divakaruni and Brand, 2011). Therefore, severe uncoupling of the mitochondrial membrane inhibits ATP synthesis and leads to cell death (Busiello et al., 2015). Interestingly, mild uncoupling induced experimentally does not affect ATP synthesis and may help with weight loss and prevention of chronic diseases such as Type 2 Diabetes Mellitus (T2DM) (Busiello et al., 2015). Transgenic mice overexpressing uncoupling proteins such as UCP1 and UCP3 are resistant against obesity and show improved insulin sensitivity when fed a high fat diet (HFD) (Costford et al., 2006; Keipert et al., 2013; Ost et al., 2014).

At high concentrations, UCB uncouples the mitochondrial membrane (Ostrow et al., 2004; Watchko and Tiribelli, 2013). However, whether mildly elevated concentrations of UCB affect mitochondrial function remains unknown and could provide a rational explanation for improved body composition and reduced risk of metabolic syndrome in individuals with increased UCB concentrations (Vitek and Ostrow, 2009; Vaz et al., 2010; Vítek, 2012; Pennell et al., 2019). Interestingly, individuals with Gilbert’s syndrome (GS) who have mildly elevated UCB concentrations (17–80 µM) are protected against cardiovascular diseases (CVDs) and have reduced body mass index (BMI) compared to individuals with normal UCB levels (<17 µM serum UCB) (Bulmer et al., 2013; Bulmer et al., 2018). Generally, the protection of GS individuals from chronic disease is attributed to the antioxidant properties of UCB (Stocker et al., 1987; Neuzil and Stocker, 1994; Bakrania et al., 2016; Bulmer et al., 2018; Shiels et al., 2019). However, few studies have explored the impact of mildly elevated concentrations of UCB on body composition and fewer still have investigated its effect on cellular and mitochondrial metabolism *in vivo* (Liu et al., 2015; Stec et al., 2016; Zelenka et al., 2016; Seyed Khoei et al., 2018).

Gunn rats have a congenital unconjugated hyperbilirubinemia in the upper range of GS due to the absence of functional UDP-glucuronosyltransferase 1A1 (UGT1A1), which leads to impaired conjugation and elimination of UCB. Therefore, these animals serve as a relevant model to study the effects of unconjugated hyperbilirubinemia on body composition and metabolism. In the present study, we investigated whether Gunn rats had reduced bodyweight, altered body composition, and impaired skeletal muscle and hepatic mitochondrial function compared to normobilirubinemic littermate rats.

## Materials and Methods

### Materials

All consumables were obtained from Sigma-Aldrich (Australia) unless otherwise stated. Chemicals for high-resolution respirometry were obtained from manufacturers as recommended by Oroboros Instruments (Fontana-Ayoub et al., 2016).

### Synthesis of Sodium Bilirubinate

Sodium bilirubinate was synthesized in order to make UCB soluble in respiration buffer (Mir06: 280 U mL^−1^ catalase, 0.5 mM EGTA, 3 mM MgCl_2_^.^6H_2_O, 60 mM K-lactobionate, 20 mM taurine, 10 mM KH_2_PO_4_, 20 mM HEPES, 110 mM sucrose, and 1 g L^−1^ bovine serum albumin (BSA), pH 7.1). 2.1 M equivalents of NaOH solution (1.5 mg ml^−1^ in ethanol) was added to UCB. The mixture was vortexed vigorously and diluted 10-fold with ethanol and evaporated in the dark under vacuum in a rotary evaporator (Maxivac; Labogene, Australia) at 21°C for 4 h. The UCB content of the powder was determined to be >99% of the commercially supplied standard, using high-performance liquid chromatography photodiode array (HPLC:PDA) as previously published (Bulmer et al., 2011).

### Animals

Breeding pairs of heterozygote and homozygote Gunn rats on a Wistar background were imported from the Rat Research and Resource Centre (Columbia, MO, United States) and bred to produce both hyperbilirubinemic (homozygote) and normobilirubinemic (heterozygote) Gunn rats. The Gunn rat phenotype was determined based on the presence of jaundice in the first 3 days after birth. Jaundiced Gunn rats were tagged, and their phenotype was confirmed by measuring TBIL concentrations. From herein, animals expressing hyperbilirubinemia are phenotypically defined as “Gunn” rats while littermates with normal bilirubin levels are termed as “normobilirubinemic controls”. Animals were housed in the G26 animal house facility at Griffith University (Gold Coast, Australia) at constant temperature (20°C) and humidity (60%), with a 12 h light: dark cycle. All animals were provided a standard rodent diet (18% Protein Rodent Diet, 18% protein, 6.2% fat; TEKLAD Standard Global, United States) and water *ad libitum.* All procedures were approved by the Griffith University Animal Ethics Research Committee (MSC/02/17/AEC) prior to experimentation.

### Experimental Protocol

Thirty six age-matched rats (∼10 weeks of age; Cohort 1) were separated into four groups based on sex and phenotype: Gunn female (*n* = 10), Control female (*n* = 9), Gunn male (*n* = 9), Control male (*n* = 8). Gunn animals were hyperbilirubinemic and were compared to normobilirubinemic controls of the same sex. These animals were gradually acclimatized in metabolic cages for 2, 5, then 24 h before entering the protocol. Acclimatized animals entered a 17-days protocol (Day 0–16) and were housed in metabolic cages three times for a period of 24 h at a time, at Day 0, 11, and 15 (11–13 weeks of age) of the protocol. In the metabolic cages, food and water remained available *ad libitum* and was weighed before and after the animal was housed to determine daily energy intake. Bodyweight was recorded every 2 days from Day 0 in addition to before and after each metabolic cage day. To control for future therapeutic interventions additional procedures (i.p. saline and p.o. water) were conducted as described in Supplementary Material (see *Additional procedures*). Energy efficiency was calculated by subtracting the final bodyweight at Day 16 from the initial bodyweight at Day 0 and this value was then divided by the calories (kcal) consumed daily $\left(Energy\;efficiency=\frac{weight\;gained\;per\;day\;(g)}{\text{calories consumed per day }(\text{kcal})}\right)$. Daily calories consumed were calculated from the average food intake across 3 metabolic cage days.

A second cohort (Cohort 2) of rats (Gunn female (*n* = 11), Control female (*n* = 11), Gunn male (*n* = 8), Control male (*n* = 7)) were bred to similar age (13–14 weeks old) and anesthetized after an overnight fast using 50 mg kg^−1^ sodium pentobarbitone (Pharmachem, Australia) via an i.p. injection. While anesthetized the animals were placed in a prone position and total body length of each animal was measured using a tape measurer from tip of the nose to the last sacral vertebrae. Anesthetized animals were euthanized by removal of the heart. Blood collected from the chest cavity was centrifuged (2000 g, 10 min, 21°C) and the serum was flash frozen and stored at −80°C for later analysis. Organs and skeletal muscle [soleus and extensor digitorum longus (EDL)] were dissected free from fat and washed in ice cold dPBS (Gibco^®^, United Kingdom), then patted dry, and weighed on a calibrated balance. Immediately after, a section of liver tissue was flash frozen and stored at −80°C for later analysis. Likewise, a piece of liver, EDL, and soleus were stored in Mir06 (liver) or BIOPS (muscle; 2.77 mM CaK_2_EGTA, 7.23 mM K_2_EGTA, 20 mM imidazole, 20 mM taurine, 50 mM MES hydrate, 0.5 mM dithiothreitol, 6.56 mM MgCl_2_ 6H_2_O, 5.77 mM Na_2_ATP, 15 mM Na_2_phosphocreatine, pH 7.1) as described in “*High-resolution respirometry*”.

Additionally, a third cohort (Cohort 3) of rats (Gunn female (*n* = 9), Control female (*n* = 10), Gunn male (*n* = 7), Control male (*n* = 17)) were bred to similar age (13–14 weeks old) and scanned using dual-energy X-ray absorptiometry (DEXA) as described in “*DEXA scan*”.

Finally, a fourth cohort (Cohort 4) of juvenile (3–4 weeks of age) female control rats (*n* = 5) was euthanized using 50 mg kg^−1^ sodium pentobarbitone i.p. following an overnight fast and a piece of liver tissue was stored in Mir06 as described in “*High-resolution respirometry*”.

### Analysis of Serum Biochemistry

Serum samples were thawed and analyzed using the COBAS Integra® 400+ (Roche Diagnostics, Australia) for direct reacting bilirubin (BILD2), TBIL (BILT3) and albumin (ALB). All assays were calibrated with appropriate standards (CFAS) and accuracy was checked with appropriate quality controls [precinorm clinchem multi 1 (PCCC1), precinorm clinchem multi 2 (PCCC2)]. All analyses were conducted in duplicate and the average of each parameter reported.

### Lipid Extraction

Frozen liver tissue (∼100 mg) was homogenized frozen (with liquid N_2_) using a mortar and pestle. Lipids were extracted with isopropyl-alcohol at a 50:1 mg: mL ratio of tissue to isopropyl-alcohol as previously published (Hsun-Wei Huang et al., 2006). Homogenates were vortexed vigorously and sonicated for 10 min and then centrifuged (43,000 g, 10 min, 21°C). The supernatant was collected in a separate tube and evaporated using a rotary evaporator at 35°C. Dry pellets were reconstituted in 250 µL of isopropanol and triglycerides were analyzed spectrophotometrically (TRIGL, Roche Diagnostics) on the COBAS Integra 400+ following manufacturer’s instruction.

### High-Resolution Respirometry

#### Liver Homogenate

A small section of liver tissue was dissected from the left lateral lobe and added directly to ice-cold Mir06 buffer. Two to three pieces weighing 8–16 mg of wet-weight (Wd) of dissected liver tissue were transferred (SHREDDER Tube, Pressure BioSciences, United States) and homogenized using a PBI shredder (Shredder SG3 Kit, Pressure BioSciences, MA, United States) as previously published (Larsen et al., 2014). Using the strongest force (position 3) the tissue was shredded for 5 s, followed by medium force (position 2) for 3 s. The homogenate was then diluted into ice-cold Mir06 to a final concentration of 1.3 mg tissue mL^−1^ and transferred into Oxygraph chambers (Oxygraph-2k; Oroboros Instruments, Austria). After O_2_ flux stabilized, the substrate-uncoupler-inhibitor titration (SUIT) protocol was utilized to evaluate mitochondrial function described under “*Measurement of mitochondrial respiration*”.

#### Permeabilization of Skeletal Muscle Fibers

Mechanical and chemical permeabilization of skeletal muscle was conducted as previously published (Liu et al., 2015). Briefly, after dissection, soleus and EDL were added to BIOPS buffer. 5–7 mg pieces of muscle tissue were used for mechanical isolation of muscle fibers using sharp round-end tweezers. After mechanical isolation, muscle fibers were permeabilized by adding saponin (final concentration 50 μg ml^−1^) to the BIOPS buffer followed by gentle agitation on an orbital shaker for 30 min on ice. Tissue was then washed in Mir06 for 10 min with gentle agitation on ice. Permeabilized muscle fibers (0.7–1.5 mg Wd) were added to each Oxygraph chamber, with analysis performed in duplicates. Samples were analyzed using two different conditions: first batch of animals (3 per group) were analyzed at air saturation (∼180 µM O_2_) while the remaining animals were super-oxygenated to 400 µM O_2_ concentration initially, and oxygen (O_2_) concentration was maintained above 250 µM using H_2_O_2_ and catalase throughout measurement (Doerrier et al., 2018). Respiration parameters did not differ between methods, therefore, all results were combined for the final analysis.

#### Measurement of Mitochondrial Respiration

Mitochondrial respiration measurements were conducted within 2 h of sample collection at 37°C using three Oxygraph units in parallel containing 2 ml of Mir06 buffer in each chamber. Data acquisition and analysis was conducted using 7.0 DatLab software (Oroboros Instruments). Standard carbohydrate SUIT protocol as previously published (Gnaiger, 2014) was utilized to measure the contribution of carbohydrates to the rate of mitochondrial O_2_ consumption (O_2_ flux: pmol s^−1^) in liver tissue and skeletal muscle of adult female and male rats (see *Cohort 2* within “*Experimental protocol*”). Additionally, the effect of UCB on hepatic mitochondrial function was determined by adding exogenous UCB (31.3, 62.5, and 125 µM) or vehicle (H_2_O; control) to Oxygraph chambers containing liver homogenate from female control juvenile rats (see *Cohort 4* within “*Experimental protocol*”). All concentrations are represented as final concentrations. The O_2_ flux of intrinsic uncoupled respiration (LEAK) was measured in the presence of 5 mM pyruvate, 1 mM malate and 10 mM glutamate (PMG). The maximal O_2_ flux of oxidative phosphorylation (OXPHOS) through complex I (CI OXPHOS) was measured by step-wise titration with 1 mM ADP in the presence of PMG. Lack of increase in O_2_ flux (<10%) after the addition of cytochrome c confirmed the integrity of the outer mitochondrial membrane. To assess CI+II convergent OXPHOS (CI+II OXPHOS), 10 mM succinate was added in the presence of PMG, cytochrome c, and saturating levels of ADP. The maximum O_2_ flux of the electron transfer system (ETS) was measured by uncoupling CI+II O_2_ flux from ATP synthesis (noncoupled state) by step-wise titration with 0.5 µM carbonyl cyanide p-trifluoromethoxyphenylhydrazone (FCCP; an extrinsic uncoupler). Noncoupled maximal respiration of complex II (CII ETS) was measured by inhibiting complex I with rotenone (0.5 µM) and residual O_2_ flux (ROX) was measured by inhibiting mitochondrial respiration with 2.5 mM antimycin (AMA). All O_2_ flux measurements were corrected for ROX and tissue mass (pmol mg^−1^ s^−1^) or citrate synthase (pmol s^−1^ ng^−1^ CS). Flux control ratios (FCRs) were calculated by normalizing the O_2_ flux of the electron pathways (i.e. LEAK, OXPHOS and CII ETS) to a common reference state (OXPHOS or ETS).

### DEXA Scan

Dual-energy X-ray absorptiometry (DEXA) (XR-36 Quickscan densitometer 4.2.4/2.3.1, software version 2.5.3a; Norland Medical Systems, Inc. United States) was conducted on a subgroup of adult Gunn and normobilirubinemic control rats (15 weeks of age). Animals were anesthetized using ketamine (50 mg kg^−1^) and xylazine (3 mg kg^−1^) mixture in a 50:30 ratio via i. p. injection and placed in a prone position in the DEXA machine. Bone mineral density (BMD), total mass, lean mass, and fat mass was collected for each animal and all scans were performed at 1.5 × 1.5 mm resolution at 60 mm s^−1^ in small animal mode.

### Citrate Synthase

Citrate synthase (CS) activity was measured as previously published (Eigentler et al., 2015). Briefly, hepatic tissue collected from Oxygraph chambers, hepatic homogenates (see Western blot analysis), or CS standard (#C3260) were mixed with 5,5′-dithiobis(2-nitrobenzoic acid) (DTNB; #D8130), acetyl CoA (#A2181) and oxaloacetic acid (#O4126) in a 96-well plate, and the reaction was monitored at 412 nm over 30 min in a spectrophotometer (ThermoFisher Scientific, Australia). All samples were analyzed in duplicate and the average absorbance was used to calculate CS activity or the amount of CS protein using the specific activity equation #6 as previously published (Eigentler et al., 2015).

### Real-Time Quantitative Polymerase Chain Reaction (qPCR)

RNA extraction, cDNA synthesis, and qPCR were conducted according to the Minimum Information for Publication of Quantitative Real-Time PCR Experiments (MIQE) guidelines (Bustin et al., 2009). Briefly, liver samples (∼10 mg) stored in RNAlater (Invitrogen, Australia) were homogenized and RNA was extracted using a RNAeasy mini kit (Qiagen, Australia) according to manufacturer’s guidelines. The RNA concentration and quality (260/280 and 260/230 nm) were measured using a Nanodrop 1,000 spectrophotometer (ThermoFisher Scientific). Complementary DNA (cDNA) was synthesized from RNA using iScript gDNA clear cDNA synthesis kit (Bio-Rad, Australia) following manufacturer’s protocol and stored at −20°C for later analysis. qPCR was conducted using KiCqStart™ predesigned primers (Sigma-Aldrich) and QuantiNova SYBR^®^ green master mix (Qiagen) on StepOne™ real-time PCR systems (Applied Biosystems, Australia) following manufacturer’s instruction (PCR initial heat activation: 2 min at 95°C; Denaturation: 40 cycles of 95°C for 5 s per cycle; Combined annealing/extension: 40 cycles of 60°C for 10 s per cycle). All data acquisition was conducted with StepOnePlus™ (v2.3). Gene expression was calculated according to the 2^−ΔΔCq^ method. *β-2-microglobulin* was stable across group and was used as the reference gene for normalizing gene expression. The list of primers is provided in the Supplementary Table S1.

### Western Blot Analysis

Frozen liver samples were homogenized by shearing tissue through 18, 21 and then 23G needles in CelLytic MT Cell Lysis buffer (2.5:50 mg/μL; #C3228) in the presence of protease inhibitor (#P8340) and phosphatase inhibitor (#ab201114, Abcam, Australia) as per manufacturer’s protocol. Tissue supernatant was generated by centrifugation (4,000 g, 10 min, 4°C) and standardized based on protein concentration using the Pierce BCA Protein Assay Kit (ThermoFisher Scientific) as per manufacturer’s protocol. Samples were prepared in Laemmli 2X buffer (#S3401) at 1:1 ratio and were heated for 30 min at 37°C (mitochondrial protein targets) or 5 min at 95°C (AMPK), prior to loading. Proteins (10–25 µg) were separated on 12% SDS-PAGE using TGX™ FastCast™ gels (#1610175, Bio-Rad). Following electrophoresis, proteins were transferred onto 0.45 µm polyvinylidene fluoride membranes (PVDF; #IPFL0010, Millipore, Australia) for 1–2 h on ice in Towbin buffer (25 mM Tris, 192 mM glycine, and 20% (v/v) methanol) for AMPK proteins or CAPS buffer (10 mM CAPS and 10% (v/v) methanol, pH 11) for mitochondrial protein targets. Following transfer, membranes were blocked with Odyssey Blocking Buffer (Millenium Science, Australia) and incubated with primary antibodies (subunits of mitochondrial respiratory complexes, #ab110413 (Abcam); p-AMPK, #ab133448 (Abcam); AMPK, #ab80039 (Abcam); GAPDH, #14C10 (Cell Signaling, Australia)) with gentle agitation overnight at 4°C. Finally, membranes were incubated with secondary antibodies (#IRDye 680 or #IRDye 800CW, LI-COR, Australia) for 1 h with gentle agitation at room temperature and visualized using Odyssey CLX (LI-COR). Densitometry analysis of mitochondrial respiratory complexes was normalized to GAPDH while p-AMPK was normalized as a ratio to total AMPK (Image Studio Lite version 5.2; LI-COR).

### Statistical Analysis

All values are expressed as mean ± (standard deviation), while multiple linear regression data are presented as un-standardized regression coefficients and 95% confidence intervals. Multicollinearity in multiple linear regression was ruled out by a variance inflation factor (VIF) of less than 5. Data was tested for normality and homogeneity of variance with Kolmogorov-Smirnov and Spearman tests, respectively. Comparisons were performed between phenotypes from the same sex using unpaired t-tests. When data failed normality or equality of variance, nonparametric Mann–Whitney test and Welch’s correction were used, respectively. The dose-dependent effect of UCB on mitochondrial respiration was compared using repeated measures two-way ANOVA with Bonferroni’s post-hoc test comparing the effect of UCB treatment to control (vehicle) on each respiratory state (e.g. CI OXPHOS and CI+II OXPHOS). Statistical analysis was performed in GraphPad PRISM (v8.2) and *p* < 0.05 was considered significant.

## Results

### Phenotype

Breeding pairs produced similar distribution of heterozygote controls (normobilirubinemic) and homozygote Gunn (hyperbilirubinemic) rats over 7 litters (Supplementary Table S2; NS). Serum TBIL concentrations were significantly greater in Gunn rats compared to controls (*p* < 0.001, Table 1).

**TABLE 1:** TBIL concentrations and body composition of hyperbilirubinemic and normobilirubinemic rats. Bold values represent statistically significant *p*-values (< 0.05). The non-bold values are non-significant *p*-values (i.e. > 0.05).

Variable	Phenotype		*P* value
	Control	Gunn	
Serum TBIL (µmol L^−1^)			
Males	2.76 (0.66)	99.8 (15.9)	**<0.001**
Females	2.33 (1.20)	79.3 (20.6)	**<0.001**
Body length (cm)			
Males	25.2 (1.10)	23.9 (1.15)	0.09
Females	21.2 (0.65)	19.7 (0.58)	**<0.001**
Bodyweight (g)			
Males	309 (17.7)	261 (37.4)	**<0.001**
Females	176 (16.5)	150 (9.57)	**<0.001**
Lean mass (g)			
Males	256 (18.4)	215 (32.1)	**<0.001**
Females	160 (16.0)	140 (12.1)	**<0.01**
Fat mass (g)			
Males	53.0 (17.7)	46.8 (19.3)	0.46
Females	16.1 (6.65)	9.94 (5.35)	**<0.05**
Liver triglycerides (mg g^−1^)			
Males	9.99 (3.16)	9.45 (4.38)	0.78
Females	4.65 (1.67)	2.39 (0.92)	**<0.01**
Lean mass (% of bodyweight)			
Males	83.0 (5.34)	82.3 (6.82)	0.79
Females	90.9 (3.81)	93.3 (3.83)	0.18
Fat mass (% of bodyweight)			
Males	17.0 (5.34)	17.7 (6.82)	0.79
Females	9.13 (3.81)	6.70 (3.83)	0.18
Soleus (mg)			
Males	175 (38.1)	134 (24.2)	**<0.05**
Females	104 (18.1)	76.3 (11.0)	**<0.001**
EDL (mg)			
Males	147 (20.5)	120 (10.1)	**<0.01**
Females	89.7 (7.80)	64.0 (9.72)	**<0.001**

Two different cohorts of animals were used for the parameters analyzed. DEXA analysis (bodyweight, lean mass, and fat mass) was conducted on animals from Cohort 3 constituting 27 control and 16 Gunn rats. All other parameters were measured on animals from Cohort 2 constituting 18 control and 19 Gunn rats. Control group represents normobilirubinemic heterozygote littermates. Gunn group represents hyperbilirubinemic homozygote littermates. TBIL, total bilirubin; EDL, extensor digitorum longus. Values are represented as mean (standard deviation). All comparisons are made between phenotype within the same sex.

### Body Composition

A total of 16 homozygote Gunn and 27 heterozygote controls were scanned using DEXA to determine body composition (Table 1). Both male and female Gunn rats had significantly lower bodyweight (*p* < 0.001) and lean mass (*p* < 0.01) when compared to controls (Table 1). Female Gunn rats had significantly lower body length (*p* < 0.001), fat mass (*p* < 0.05), and liver triglyceride concentration (*p* < 0.01) compared to female controls (Table 1). Male and female Gunn rats had significantly lower soleus and EDL muscle mass compared to littermate controls (*p* < 0.01; Table 1).

### Organ Weights

There were no significant differences in liver mass between groups (Table 2). Heart and lung mass were only significantly reduced (*p* < 0.001) in female Gunn rats when compared to controls, with a trend toward reduced heart (*p* = 0.07) and lung (*p* = 0.14) mass in male Gunn rats (Table 2). Only female Gunn rats had significantly reduced kidney mass (*p* < 0.001), however, all Gunn rats presented a trend toward a reduced spleen mass (*p* = 0.15 and *p* = 0.05, respectively) compared to controls (Table 2). Additional organ masses are presented in Table 2. Organ masses expressed relative to bodyweight are provided in Supplementary Table S3.

**TABLE 2:** Organ weights of hyperbilirubinemic and normobilirubinemic rats. Bold values represent statistically significant *p*-values (< 0.05). The non-bold values are non-significant *p*-values (i.e. > 0.05).

Variable	Phenotype		*P* value
	Control (*n* = 18)	Gunn (*n* = 19)	
Liver (g)			
Males	14.8 (3.01)	13.8 (2.79)	0.52
Females	8.84 (1.23)	8.74 (0.91)	0.82
Heart (g)			
Males	1.19 (0.11)	1.07 (0.13)	0.07
Females	0.82 (0.06)	0.67 (0.06)	**<0.001**
Lungs (g)			
Males	1.54 (0.23)	1.37 (0.19)	0.14
Females	1.15 (0.12)	0.95 (0.08)	**<0.001**
Kidney (g)			
Males	1.62 (0.23)	1.53 (0.22)	0.48
Females	0.98 (0.06)	0.77 (0.04)	**<0.001**
Spleen (g)			
Males	1.17 (0.12)	1.03 (0.13)	**0.05**
Females	0.80 (0.10)	0.73 (0.12)	0.15
Testes (g)			
Males	1.89 (0.20)	1.75 (0.13)	0.24

Control group represents normobilirubinemic heterozygote littermates. Gunn group represents hyperbilirubinemic homozygote littermates. Values are represented as mean (standard deviation). All comparisons are made between phenotype within the same sex.

### Multiple Linear Regression of Organ Weights

In order to differentiate the effects of the hyperbilirubinemic phenotype (condition) from bodyweight on organ mass, multiple linear regression was conducted with organ mass as a dependent variable while bodyweight and the presence/absence of hyperbilirubinemia were used as independent variables. In male rats, bodyweight significantly and positively predicted all organ and skeletal muscle weights (*p* < 0.01) while the hyperbilirubinemic phenotype was a significant negative predictor of soleus and EDL mass (*p* < 0.05, Table 3). In females, bodyweight and the hyperbilirubinemic phenotype significantly and positively predicted liver mass (*p* < 0.001), however, only the hyperbilirubinemic phenotype was a significant negative predictor of kidney mass (*p* < 0.01, Table 3). Additionally, bodyweight was a significant positive predictor of soleus and EDL mass in females (*p* < 0.05. Table 3). In the second analysis, data from both sexes were combined, which demonstrated that bodyweight was a significant positive predictor for all organ and skeletal muscle masses (*p* < 0.001, Table 3). Conversely, the hyperbilirubinemic phenotype was a significant positive predictor for liver and kidney mass (*p* < 0.001), and a negative predictor for soleus and EDL mass (*p* < 0.05, Table 3).

**TABLE 3:** Multiple linear regression of the effect of bodyweight and the hyperbilirubinemic phenotype on organ weights and skeletal muscle. Bold values represent statistically significant *p*-values (< 0.05). The non-bold values are non-significant *p*-values (i.e. > 0.05).

	Independent variables			
	Bodyweight^a^ B_1_ (95% CI)	Phenotype^b^ B_2_ (95% CI)	*R* ^2^	*P* value
	
Dependent variables				
Liver (mg)				
Males (*n* = 14)	40.7 (32.0, 49.3)	1,070 (−156, 2,300)	0.91	**<0.001** ^a^, 0.08^b^
Females (*n* = 22)	35.6 (21.0, 50.2)	2,440 (1,220, 3,670)	0.58	**<0.001** ^a^, **<0.001** ^b^
Combined (*n* = 36)	32.0 (28.7, 35.3)	1,550 (873, 2,230)	0.92	**<0.001** ^a^ **, <0.001** ^b^
Heart (mg)				
Males (*n* = 14)	1.69 (1.20, 2.18)	−31.8 (−101, 37.6)	0.87	**<0.001** ^a^, 0.33^b^
Females (*n* = 20)	0.90 (−0.39, 2.20)	−77.5 (−185, 29.9)	0.64	0.16^a^, 0.15^b^
Combined (n = 34)	1.99 (1.78, 2.20)	−4.21 (−47.3, 38.8)	0.93	**<0.001** ^a^, 0.84^b^
Lungs (mg)				
Males (*n* = 14)	2.78 (1.88, 3.68)	−34.9 (−162, 92.6)	0.84	**<0.001** ^a^, 0.56^b^
Females (*n* = 21)	1.63 (−0.38, 3.63)	−80.6 (−253, 91.7)	0.58	0.11^a^, 0.34^b^
Combined (*n* = 35)	2.31 (1.98, 2.64)	−43.5 (−112, 24.8)	0.88	**<0.001** ^a^, 0.20^b^
Kidney (mg)				
Males (*n* = 14)	2.86 (1.78, 3.94)	49.9 (−104, 203)	0.77	**<0.001** ^a^, 0.49^b^
Females (*n* = 22)	0.82 (−0.12, 1.76)	−147 (−226, −67.6)	0.85	0.08^a^, **<0.01** ^b^
Combined (*n* = 36)	3.62 (3.27, 3.96)	71.1 (0.29, 142)	0.93	**<0.001** ^a^, **<0.05** ^b^
Spleen (mg)				
Males (*n* = 14)	1.37 (0.50, 2.23)	−56.7 (−180, 66.4)	0.63	**<0.01** ^a^, 0.33^b^
Females (*n* = 22)	1.67 (−0.45, 3.79)	49.6 (−128, 228)	0.21	0.12^a^, 0.57^b^
Combined (*n* = 36)	1.70 (1.36, 2.04)	18.4 (−52.3, 89.1)	0.76	**<0.001** ^a^, 0.60^b^
Soleus (mg)				
Males (*n* = 14)	0.44 (0.33, 0.54)	−15.9 (−31.1, −0.66)	0.92	**<0.001** ^a^, **<0.05** ^b^
Females (*n* = 22)	0.46 (0.24, 0.68)	5.10 (−13.3, 23.5)	0.74	**<0.001** ^a^, 0.57^b^
Combined (*n* = 36)	0.36 (0.32, 0.40)	−9.22 (−17.9, −0.57)	0.91	**<0.001** ^a^, **<0.05** ^b^
EDL (mg)				
Males (*n* = 14)	0.19 (0.10, 0.27)	−14.9 (−27.3, −2.46)	0.81	**<0.001** ^a^, **<0.05** ^b^
Females (*n* = 22)	0.21 (0.05, 0.36)	−11.1 (−24.0, 1.85)	0.79	**<0.05** ^a^, 0.09^b^
Combined (*n* = 36)	0.28 (0.25, 0.31)	−6.89 (−13.5, −0.33)	0.92	**<0.001** ^a^, **<0.05** ^b^

Phenotype was entered as a dummy variable with normobilirubinemic phenotype used as the reference group to which the hyperbilirubinemic phenotype was compared. B_1-2_ represent the un-standardized regression coefficients for bodyweight and phenotype, respectively. The “B coefficient” estimates the change in mass (mg) of the dependent variables per unit of increase in bodyweight (g) or in the presence of the hyperbilirubinemic phenotype. CI, Confidence Interval; EDL, extensor digitorum longus.

^a^ Statistical significance of bodyweight as an independent predictor of organ weight *p* < 0.05.

^b^ Statistical significance of phenotype as an independent predictor of organ weight *p* < 0.05.

### Energy Balance

Body mass and food intake were measured over a 17-day period. Female Gunn rats gained significantly less bodyweight and consumed significantly fewer energy daily compared to controls (*p* < 0.01, Table 4). In contrast, there was no difference in bodyweight gain and food intake in male Gunn rats compared to controls (Table 4). Bodyweight gain relative to energy intake (energetic efficiency) was significantly reduced in female Gunn rats compared to controls (*p* < 0.05), however, no difference existed in males (Table 4).

**TABLE 4:** Food intake, bodyweight gain, and energy efficiency of hyperbilirubinemic rats and their normobilirubinemic littermates. Bold values represent statistically significant *p*-values (< 0.05). The non-bold values are non-significant *p*-values (i.e. > 0.05).

Variable	Phenotype		*P* value
	Control	Gunn	
Food intake (kcal day^−1^)			
Males	83.5 (14.1)	82.4 (6.78)	0.85
Females	63.3 (6.95)	54.1 (6.38)	**<0.01**
Bodyweight gain (Day 16–Day 0) (g)			
Males	22.1 (4.78)	21.1 (14.1)	0.84
Females	15.2 (5.29)	6.60 (6.10)	**<0.01**
Energy efficiency (mg kcal^−1^)			
Males	15.6 (2.70)	17.0 (7.60)	0.65
Females	14.9 (5.10)	8.09 (5.75)	**<0.05**

Control and Gunn groups represent normobilirubinemic heterozygote and hyperbilirubinemic homozygote littermates, respectively. Values are represented as mean (standard deviation). Statistical comparisons are made between phenotypes within the same sex.

### Liver and Skeletal Muscle Mitochondrial Function

**FIGURE 1:**
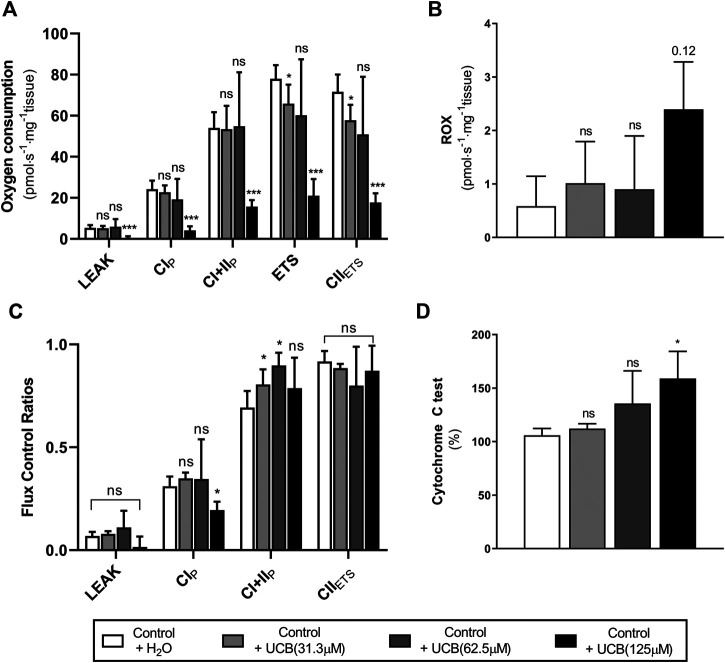
Effect of exogenous UCB on mitochondrial function in juvenile control (normobilirubinemic) liver tissue (n = 5). Mitochondrial respiratory states are identified as intrinsic uncoupling measured in the absence of ADP (LEAK), OXPHOS capacity measured at saturating levels of ADP via CI (CI_P_) or CI+CII (CI+II_P_) and noncoupled respiratory capacity (ETS and CII_ETS_). **(A, B)** The rate of respiratory states evaluated based on O_2_ consumption per mass of tissue at varying UCB concentrations. **(C)** Respiratory states expressed relative to a common reference state (ETS). **(D)** Evaluating mitochondrial outer membrane integrity by the addition of cytochrome c, represented as % change in O_2_ consumption relative to before cytochrome c addition. CI, CII, mitochondrial respiratory chain Complex I and Complex II, respectively; ETS, electron transfer system; ROX, residual O_2_ consumption; UCB, unconjugated bilirubin. *p* < 0.05*, <0.01**, <0.001*** compared to control+H_2_O. ns: non-significant.

Given that reduced energetic efficiency was reported in female Gunn rats (Table 4), the effect of acute UCB on mitochondrial function was investigated *in vitro*. Exogenous UCB was added to female control (normobilirubinemic) liver tissue and the dose-dependent effect of UCB on mitochondrial function was assessed (Figure 1). At the highest concentration (125 µM), UCB significantly inhibited LEAK, CI and CI+II OXPHOS respiration when compared to control (*p* < 0.001 and *p* < 0.05, respectively; Figure 1A). Furthermore, 31.3 and 125 µM UCB significantly inhibited CI+II ETS and CII ETS compared to control (*p* < 0.01 and *p* < 0.001, respectively; Figure 1A). The flux control ratio (FCR) of CI+II OXPHOS was significantly reduced by 31.3 and 62.5 µM UCB compared to control (*p* < 0.05 and *p* < 0.01, respectively; Figure 1C). O_2_ flux following cytochrome c addition was significantly increased with 125 µM UCB compared to control (*p* < 0.05; Figure 1D).

**FIGURE 2:**
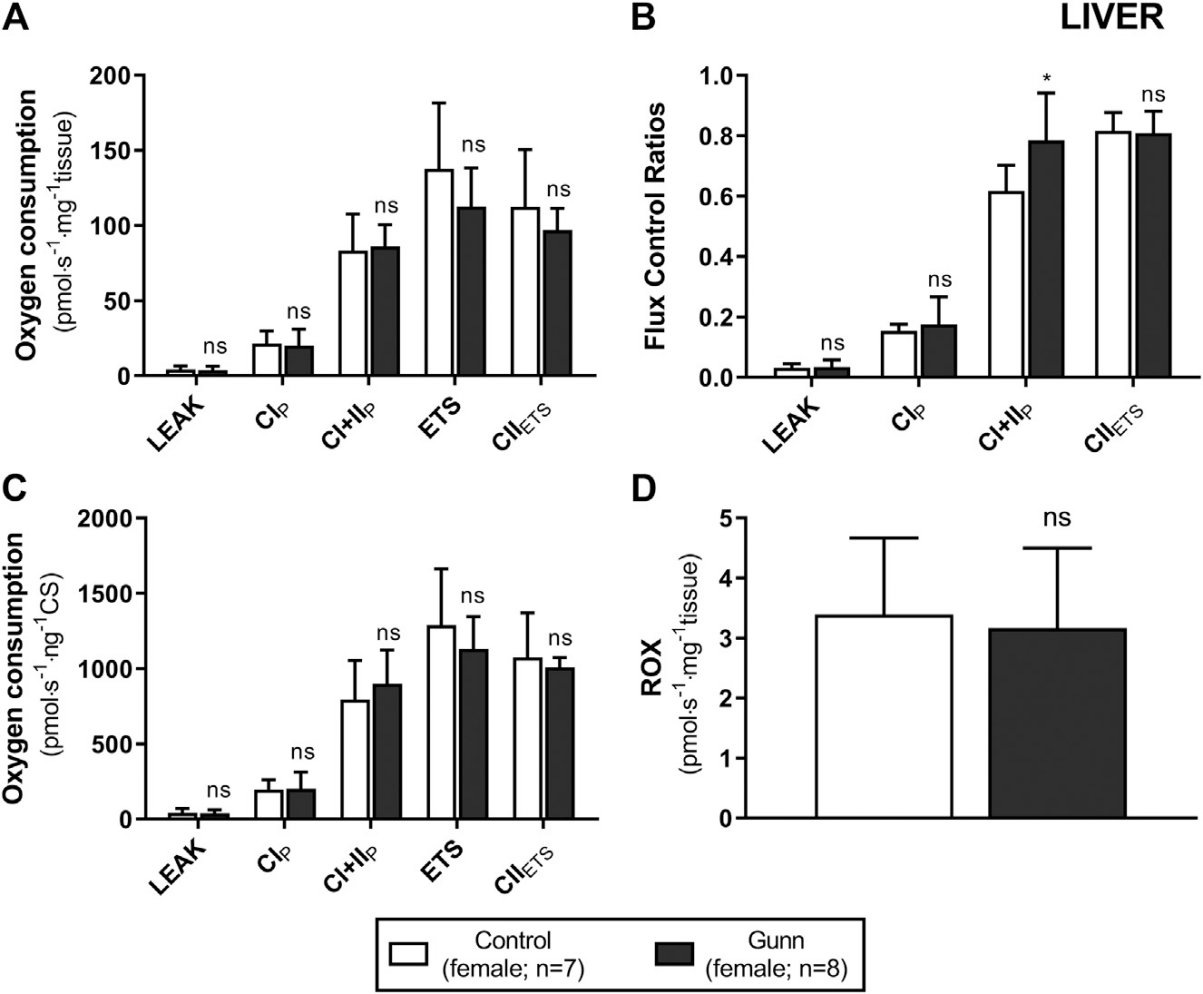
Mitochondrial function in adult female Gunn (hyperbilirubinemic) and control (normobilirubinemic) liver tissue. Mitochondrial respiratory states are identified as intrinsic uncoupling measured in the absence of ADP (LEAK), OXPHOS capacity measured at saturating levels of ADP via CI (CI_P_) or CI+CII (CI+II_P_) and noncoupled respiratory capacity (ETS and CII_ETS_). **(A, D)** The rate of respiratory states evaluated based on O_2_ consumption per mass of tissue. **(B)** Respiratory states expressed relative to a common reference state (ETS). **(C)** The rate of respiratory states evaluated based on O_2_ consumption per protein of citrate synthase. CI, CII, mitochondrial respiratory chain Complex I and Complex II, respectively; ETS, electron transfer system; ROX, residual O_2_ consumption. *p* < 0.05*, <0.01**, <0.001*** compared to control. ns: non-significant.

**FIGURE 3:**
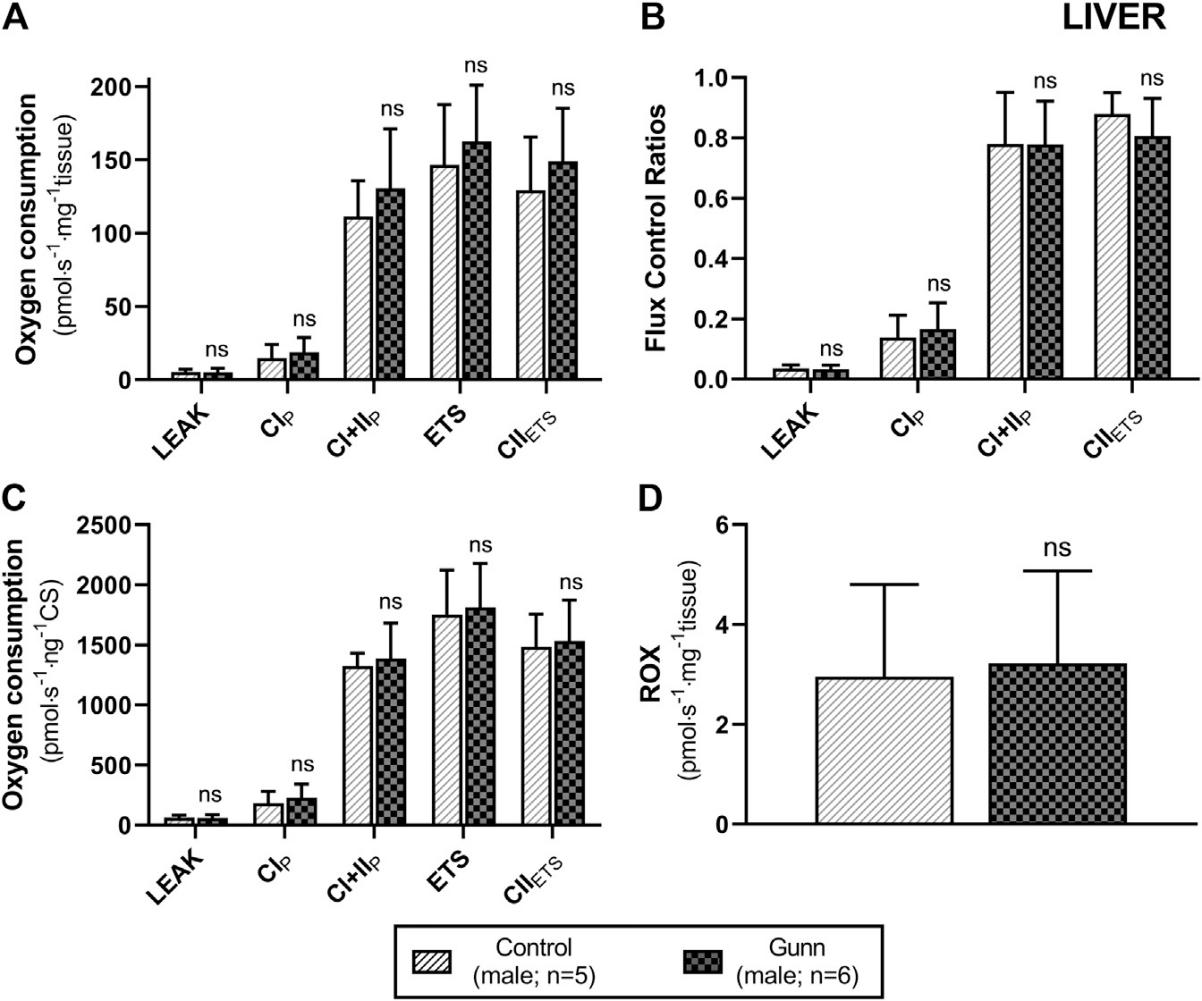
Mitochondrial function in adult male Gunn (hyperbilirubinemic) and control (normobilirubinemic) liver tissue. Mitochondrial respiratory states are identified as intrinsic uncoupling measured in the absence of ADP (LEAK), OXPHOS capacity measured at saturating levels of ADP via CI (CI_P_) or CI+CII (CI+II_P_) and noncoupled respiratory capacity (ETS and CII_ETS_). **(A, D)** Respiratory states evaluated based on O_2_ consumption per mass of tissue. **(B)** The rate of respiratory states expressed relative to a common reference state (ETS). **(C)** The rate of respiratory states evaluated based on O_2_ consumption per protein of citrate synthase. CI, CII, mitochondrial respiratory chain Complex I and Complex II, respectively; ETS, electron transfer system; ROX, residual O_2_ consumption. *p* < 0.05*, <0.01**, <0.001*** compared to control. ns: non-significant.

To assess whether similar effects observed *in vitro* with exogenous UCB were recapitulated in adult hyperbilirubinemic Gunn rats, mitochondrial function was assessed in fresh liver tissue. No significant change in O_2_ flux (LEAK, OXPHOS, or ETS) was found between Gunn rats and controls, when normalized to liver mass or citrate synthase activity (Figures 2, 3). However, CI+II OXPHOS FCR was significantly increased in female Gunn rats compared to controls (*p* < 0.05; Figure 2B).

**FIGURE 4:**
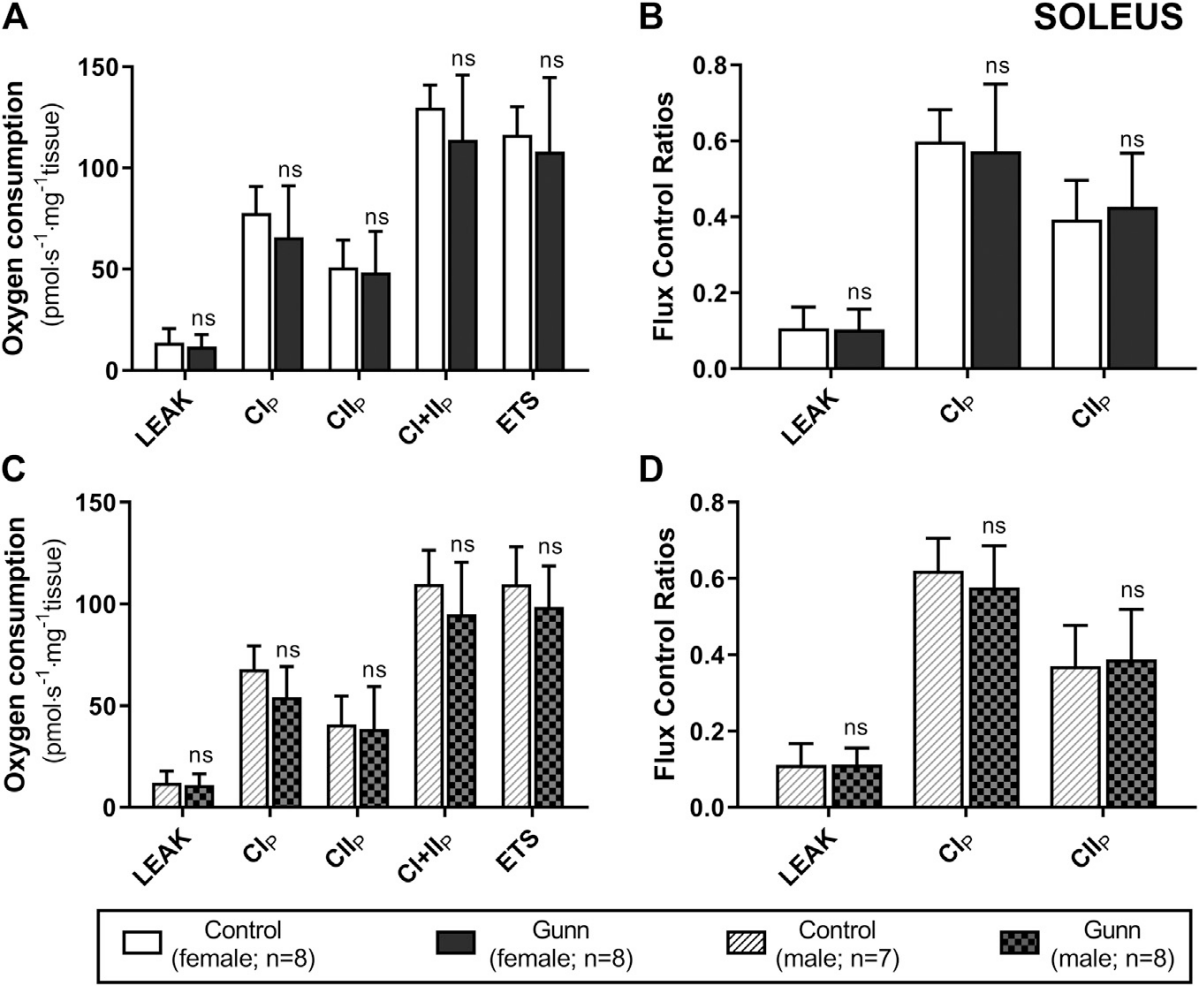
Mitochondrial function in adult female **(A, B)** and male **(C, D)** Gunn (hyperbilirubinemic) and control (normobilirubinemic) permeabilized soleus fibers. Mitochondrial respiratory states are identified as intrinsic uncoupling measured in the absence of ADP (LEAK), OXPHOS capacity measured at saturating levels of ADP via CI (CI_P_), CII (CII_P_), and CI+II (CI+II_P_), and noncoupled respiratory capacity (ETS). **(A, C)** The rate of respiratory states evaluated based on O_2_ consumption per mass of tissue. **(B, D)** Respiratory states expressed relative to a common reference state (CI+II_P_). CI, CII, mitochondrial respiratory chain Complex I and Complex II, respectively; ETS, electron transfer system; *p* < 0.05*, <0.01**, <0.001*** compared to control. ns: non-significant.

**FIGURE 5:**
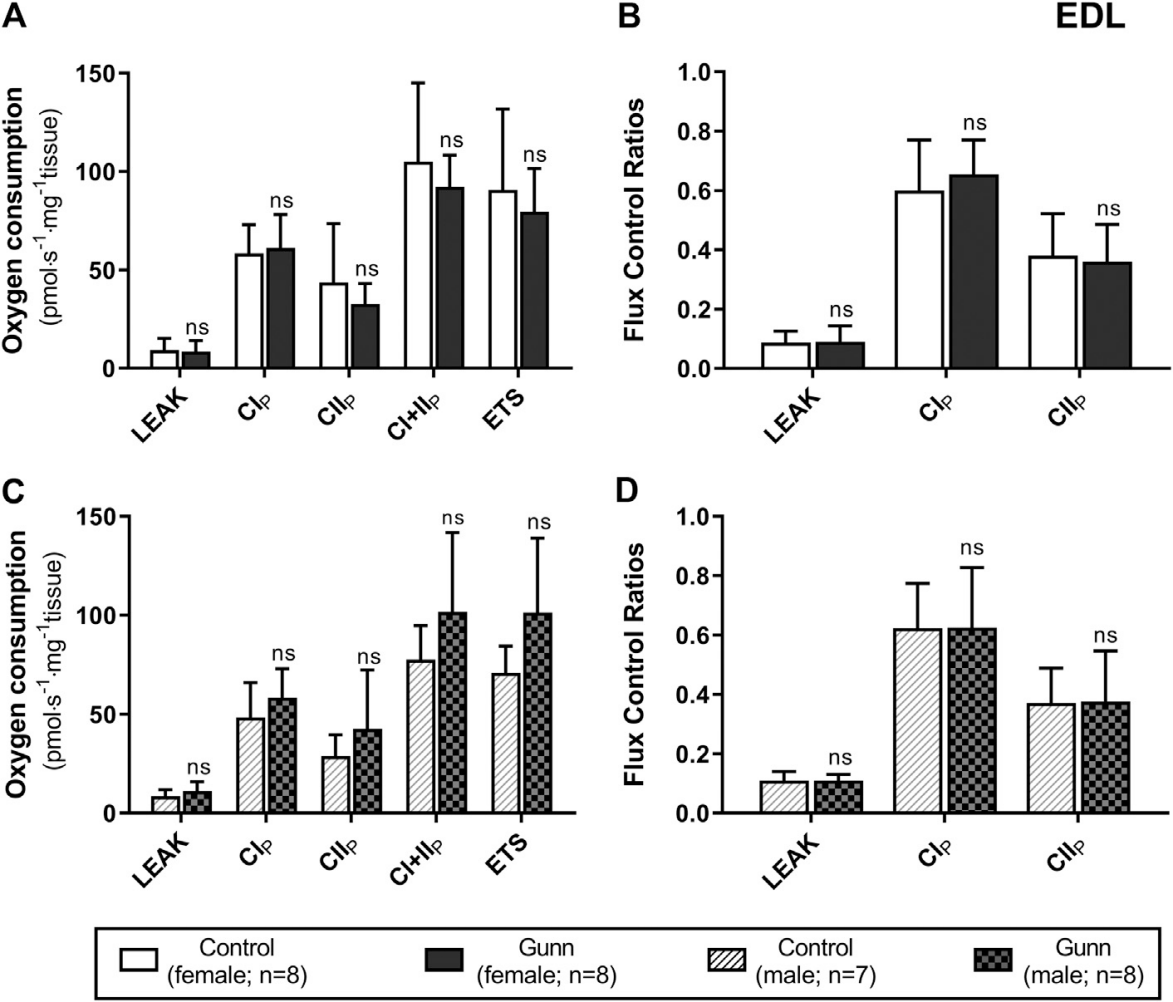
Mitochondrial function in adult female **(A, B)** and male **(C, D)** Gunn (hyperbilirubinemic) and control (normobilirubinemic) permeabilized EDL fibers. Mitochondrial respiratory states are identified as intrinsic uncoupling measured in the absence of ADP (LEAK), OXPHOS capacity measured at saturating levels of ADP via CI (CI_P_), CII (CII_P_), and CI+II (CI+II_P_), and noncoupled respiratory capacity (ETS). **(A, C)** The rate of respiratory states evaluated based on O_2_ consumption per mass of tissue. **(B, D)** Respiratory states expressed relative to a common reference state (CI+II_P_). CI, CII, mitochondrial respiratory chain Complex I and Complex II, respectively; ETS, electron transfer system; EDL, Extensor digitorum longus. *p* < 0.05*, <0.01**, <0.001*** compared to control. ns: non-significant.

There was no difference in O_2_ flux (LEAK, OXPHOS, or ETS) between Gunn and control rats for soleus or EDL (Figures 4, 5). Since CI+II OXPHOS represented the maximum O_2_ flux (greater than ETS) in skeletal muscle, CI+II OXPHOS instead of ETS was used as a reference state to calculate FCRs. FCRs were not significantly different across groups in soleus or EDL (Figures 4, 5). Gunn rats did not significantly differ in O_2_ flux after addition of cytochrome c compared to controls in liver or skeletal muscle (data not shown).

### Gene Expression Related to Fatty Acid Metabolism and PPARα Activity

**FIGURE 6:**
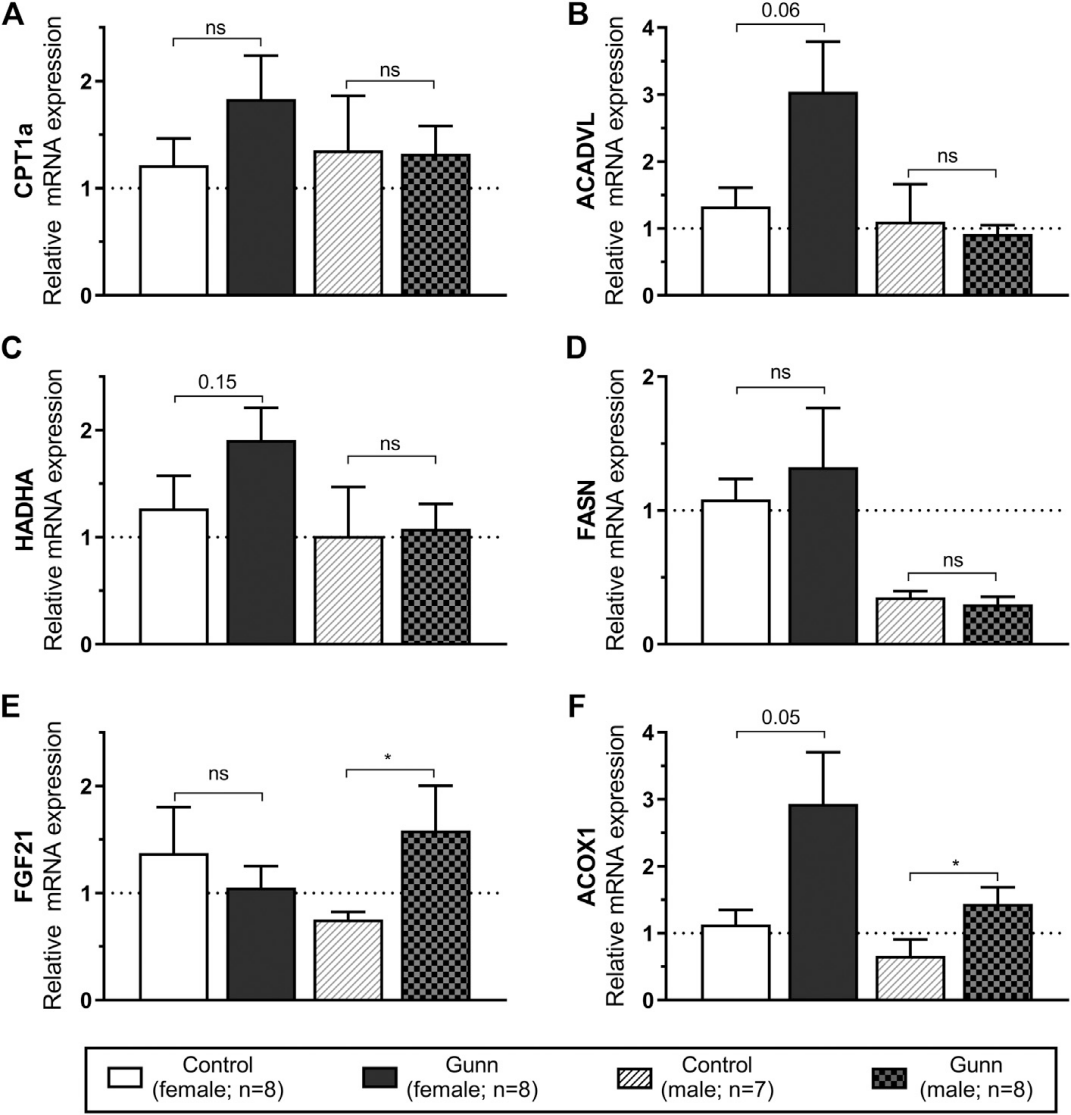
Hepatic gene expression related to fatty acid metabolism and PPARα activity in adult Gunn (hyperbilirubinemic) and control (normobilirubinemic) rats. Data are presented as mean ± standard error of the mean (SEM). *CPT1a*, carnitine palmitoyltransferase 1A; *ACADVL*, acyl-CoA dehydrogenase, very long chain; *HADHA*, hydroxyacyl-CoA dehydrogenase trifunctional multienzyme complex subunit alpha; *FASN*, fatty acid synthase; *FGF21*, fibroblast growth factor 21; *ACOX1*, acyl-CoA oxidase 1. *p* < 0.05*, <0.01**, <0.001*** compared to control. ns: non-significant.

Genes related to mitochondrial β-oxidation [Acyl-CoA dehydrogenase, very long chain (*ACADVL*) and hydroxyacyl-CoA dehydrogenase trifunctional multienzyme complex subunit alpha (*HADHA*)], fatty acid transport [carnitine palmitoyltransferase 1A (*CPT1a*)], fatty acid synthesis [fatty acid synthase (*FASN*)] and peroxisome proliferator-activated receptor alpha (*PPARα*) activity [fibroblast growth factor 21 (*FGF21*) and acyl-CoA oxidase 1 (*ACOX1*)] were measured using qPCR in hepatic tissue. *ACADVL* and *HADHA* showed a non-significant (*p* = 0.06 and *p* = 0.15, respectively; Figures 6B,C) trend toward increased expression in female Gunn compared to control rats. There was no difference in gene expression related to fatty acid metabolism between male Gunn and control rats (Figures 6A–D). *ACOX1* expression tended to increase (*p* = 0.05) while *FGF21* was not different in female Gunn vs. control rats (Figures 6E,F). In contrast, expression of *FGF21* and *ACOX1* were significantly greater in male Gunn compared to control rats (*p* < 0.05; Figures 6E,F).

### Gene Expression Related to Mitochondrial Biogenesis

**FIGURE 7:**
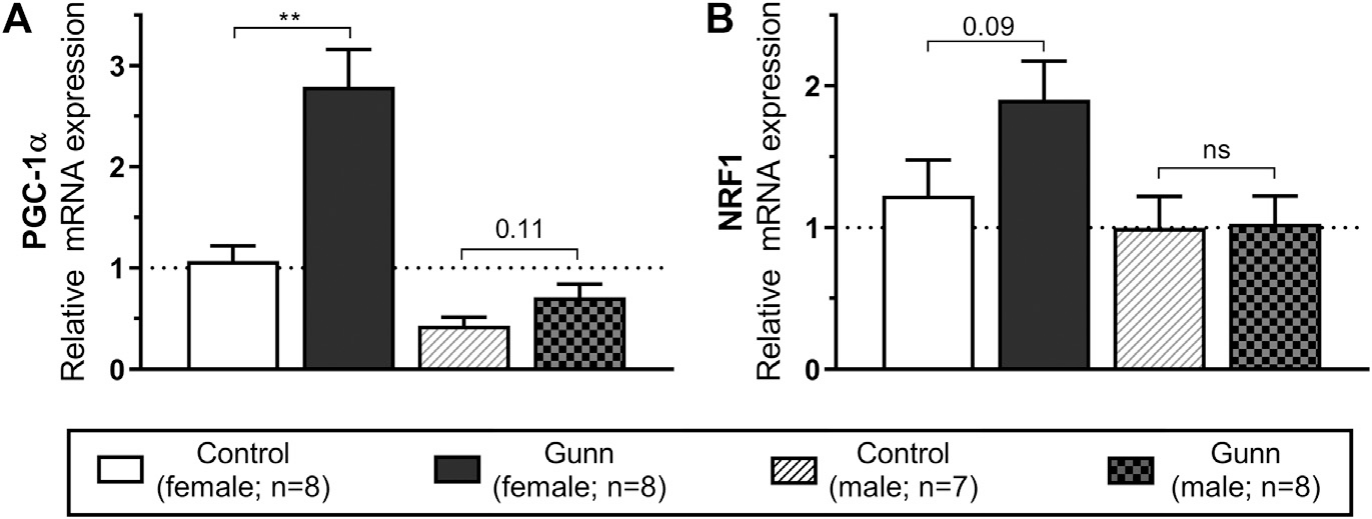
Hepatic gene expression related to mitochondrial biogenesis in adult Gunn (hyperbilirubinemic) and control (normobilirubinemic) rats. Data are presented as mean ± standard error of the mean (SEM). *PGC-1α*, peroxisome proliferative activated receptor gamma coactivator 1 alpha; *NRF1*, nuclear respiratory factor 1. *p* < 0.05*, <0.01**, <0.001*** compared to control. ns: non-significant.

Genes that regulate mitochondrial biogenesis were measured using qPCR in hepatic tissue. Peroxisome proliferative activated receptor gamma coactivator 1 alpha (*PGC-1α*) expression was significantly (*p* < 0.01; Figure 7A) increased with nuclear respiratory factor 1 (*NRF1*) tending to increase (*p* = 0.09; Figure 7B) in female Gunn vs. control rats. In comparison, *PGC-1α* tended to increase (*p* = 0.11; Figure 7A) with no change in *NRF1* (Figure 7B) in male Gunn compared to control rats.

### Expression of Subunits Specific to Hepatic Mitochondrial Respiratory Complexes

**FIGURE 8:**
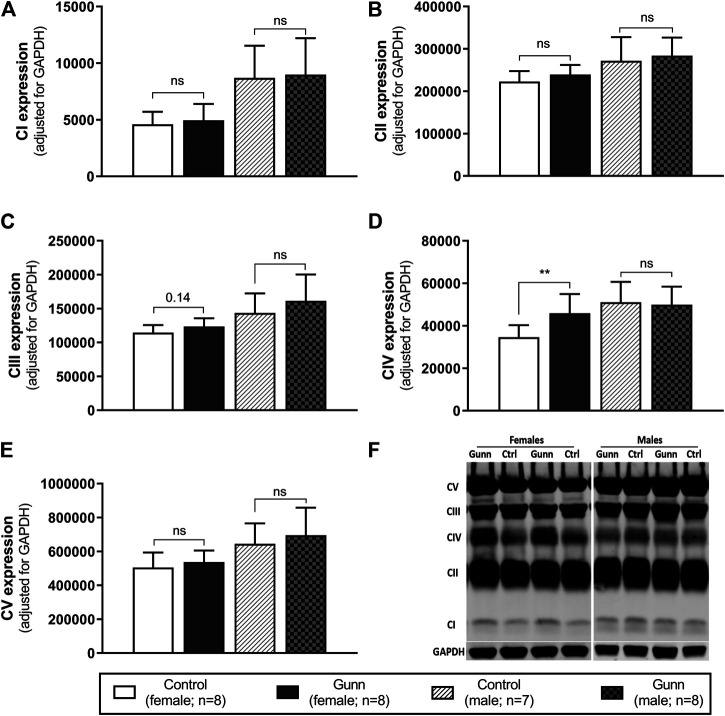
Mitochondrial quality and density assessed by western blot in adult Gunn (hyperbilirubinemic) and control (normobilirubinemic) liver tissue. **(A–E)** Protein extracts from liver tissue were investigated for protein expression of subunits representing mitochondrial respiratory complexes (CI-V) adjusted for a loading control (GAPDH). **(F)** Example western blot analysis of subunits of mitochondrial complexes. CI, complex I subunit NDUFB8; CII, complex II subunit SDHB; CIII, complex III core protein 2; CIV, complex IV subunit 1; CV, complex V subunit alpha. *p* < 0.05*, <0.01**, <0.001*** compared to control. ns: non-significant.

Finally, hepatic mitochondrial quantity and quality were evaluated by measuring the relative expression of subunits specific to mitochondrial respiratory complexes (complex I (CI) subunit NDUFB8, complex II (CII) subunit SDHB, complex III (CIII) core protein 2, complex IV (CIV) subunit 1, complex V (CV) subunit alpha) using western blot. There was significantly greater expression of CIV subunit 1 in female Gunn rats when compared to controls (*p* < 0.01; Figure 8D). No significant differences in the expression of subunits specific to mitochondrial complexes were observed in male Gunn vs. control rats (Figure 8).

### Hepatic Citrate Synthase Activity and AMPK Expression

**FIGURE 9:**
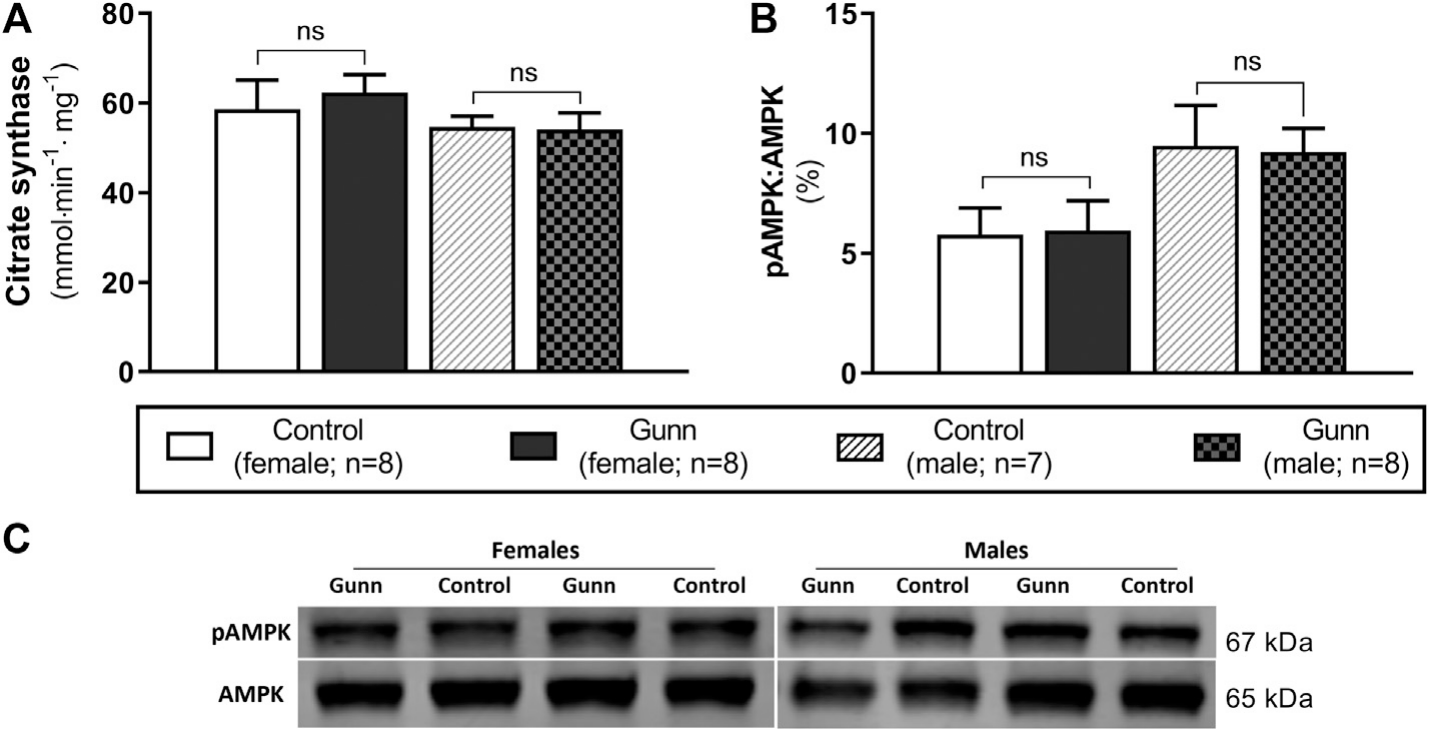
Hepatic mitochondrial density measured by citrate synthase activity and energetic state assessed using pAMPK:AMPK ratios in adult Gunn (hyperbilirubinemic) and control (normobilirubinemic) liver tissue. **(A)** Citrate synthase activity measured in protein extracts from liver tissue standardized for total protein. **(B)** Phosphorylated AMPK expression over total AMPK measured using Western blot. AMPK, AMP-activated protein kinase; pAMPK, phosphorylated AMPK. *p* < 0.05*, <0.01**, <0.001*** compared to control. ns: non-significant.

Citrate synthase activity was measured as a crude indicator of mitochondrial density in liver homogenates of adult Gunn and control rats. There were no differences in citrate synthase activity between groups (Figure 9A). Phosphorylated AMP-activated protein kinase (pAMPK) relative to total AMPK was measured as an indirect marker of the AMP to ATP ratios and the energetic state of liver tissue. Ratios of pAMPK:AMPK were unchanged across groups (Figure 9B).

## Discussion

This study presents the first comprehensive evaluation of body composition and mitochondrial function in an animal model of UGT1A1 mutation and unconjugated hyperbilirubinemia. Greater hepatic CI+II OXPHOS FCR, increased *PGC-1α* and CIV subunit 1 expression were observed in female Gunn rats, suggesting an increase in mitochondrial biogenesis. These findings were associated with lower fat mass and a trend toward increased hepatic mitochondrial β-oxidation gene expression in female Gunn rats. In contrast, exogenous addition of UCB inhibited mitochondrial function, thus, the lack of mitochondrial dysfunction in female Gunn rats may reflect the differences in bilirubin: albumin molar ratios between the two conditions and/or a compensatory mechanism to UCB inhibition. Female Gunn rats had physiological changes at both the whole body (e.g. fat mass) and subcellular (e.g. increased mitochondrial biogenesis) levels; in contrast, we did not observe many of these changes in male Gunn rats, suggesting that reduced UGT1A1 function leads to sexually dimorphic effects on metabolism.

Although previous papers have reported reduced bodyweight in hyperbilirubinemic Gunn rats compared to normobilirubinemic littermates, none have characterized the body composition of these animals (Fu et al., 2010; Boon et al., 2012; Wallner et al., 2013). Significant decrease in lean mass was observed in male and female Gunn rats compared to normobilirubinemic littermates (controls). However, significantly lower total fat mass and body length were only observed in female Gunn rats. Excess body fat relative to bodyweight [fat mass (%)] increases the risk of CVD (Zeng et al., 2012), with female Gunn rats demonstrating a notable non-significant, 26% reduction in fat mass (%) compared to controls (Table 1). Therefore, the change in female Gunn rats may be protective against CVD and this is consistent with epidemiological evidence that reports reduced risk from CVD in individuals with mildly elevated circulating UCB concentrations (Bulmer et al., 2018).

Considering that female Gunn rats had reduced fat mass we investigated whether there were changes to hepatic gene expression related to fatty acid metabolism. Interestingly, there was a notable trend toward increased expression in *ACADVL* and *HADHA* (*p* < 0.15; Figure 6) in female Gunn rats. These data suggest that mitochondrial β-oxidation could be upregulated in female Gunn rats leading to increased fat breakdown (Nguyen et al., 2008). Collectively, increased mitochondrial β-oxidation may account for the reduced energetic efficiency and decreased fat mass in female Gunn rats (Nguyen et al., 2008). On the contrary, there was no change in genes associated with fatty acid metabolism in male Gunn rats which is consistent with the lack of difference in adiposity when compared to male normobilirubinemic controls.

Stec et al. reported that a week of UCB treatment in male normobilirubinemic mice fed a HFD resulted in reduced bodyweight and fat mass (%), with increased lean mass (%) (Stec et al., 2016). Similarly, male mice with chronic hyperbilirubinemia consuming a HFD demonstrated reduced bodyweight, fat mass (%), and liver triglycerides, with increased β-oxidation gene expression when compared to normobilirubinemic controls (Hinds et al., 2017). Interestingly, these studies reported that UCB is an endogenous agonist of PPARα with the lipid-reducing effects of UCB being mediated through this nuclear receptor (Stec et al., 2016; Hinds et al., 2017). Therefore, these studies suggest that UCB is an endogenous regulator of fat metabolism and support the present results of reduced fat mass and elevated β-oxidation gene expression in a rat model of hyperbilirubinemia.

To determine whether PPARα activity was also increased in Gunn rats we investigated the expression of downstream gene targets *ACOX1* and *FGF21* (Rakhshandehroo et al., 2010). Although *ACOX1* expression was non-significantly (*p* = 0.05; Figure 6) three-fold greater in female Gunn rats there was no change in *FGF21* expression. In comparison, *ACOX1* and *FGF21* gene expression were significantly upregulated in male Gunn rats (Figure 6). These data suggest that PPARα activity is potentially increased in male but not in female Gunn rats. It is unknown whether Stec and colleagues (Stec et al., 2016; Hinds et al., 2017) found similar sexual dimorphism in PPARα activation in hyperbilirubinemic animals because their findings were reported in male mice. However, studies investigating the effectiveness of PPARα agonists such as fenofibrate demonstrated diminished activity of this receptor in ovary intact and ovariectomized mice treated with estrogen. These mice also demonstrated decreased *ACOX1* expression and attenuated fatty acid oxidation compared to estrogen poor ovariectomized mice (Yoon et al., 2002; Jeong and Yoon, 2007). Therefore, discrepancy in PPARα activity between male and female Gunn rats could potentially be determined by sexual dimorphism in the function of this receptor.

BMR contributes ∼65% of daily energy expenditure, which is proportional to lean mass and its composition (Dulloo et al., 2010). Although organs constitute a small fraction of lean mass, they determine the majority of BMR (70–90% organs vs 13–20% skeletal muscle) (Holliday et al., 1967; Even et al., 2001; Kummitha et al., 2014). In mice, the liver alone accounts for 50% of BMR while constituting only 6.2% of bodyweight (Kummitha et al., 2014). Intriguingly, organs from female Gunn rats were generally lighter than controls, however, when corrected for bodyweight were significantly heavier (Table 2 and Supplementary Table S3). Toxicity studies show that the relationship of organ mass to bodyweight is nonlinear for some organs, thus, correction for bodyweight can introduce error and misrepresent the effect of the condition/treatment on organ masses (Bailey et al., 2004). To avoid this limitation, we applied multiple linear regression to separate the effects of bodyweight from phenotype on organ masses. This analysis revealed that on average the liver mass was heavier by 2.44 g while kidney mass was lighter by 0.15 g in female Gunn rats independent of bodyweight (Table 3). This is the first study to demonstrate an association of hyperbilirubinemia/UGT1A1 impairment with organ masses. Considering that organs are more metabolically active than other forms of lean mass, larger liver mass in female Gunn rats may indicate greater BMR in these animals which would in turn explain their reduced energetic efficiency and decreased bodyweight (Table 1 and 4) (Holliday et al., 1967; Even et al., 2001; Kummitha et al., 2014). Interestingly, Hinds et al. reported that the daily rate of O_2_ consumption (VO_2_) was not different in male hyperbilirubinemic mice compared to controls, however, it remains to be investigated in female hyperbilirubinemic animals (Hinds et al., 2017). Higher voluntary physical activity is another possible reason for the leaner phenotype in Gunn rats. This conclusion is supported by data showing that 3–4 month old male Gunn rats had reduced bodyweight and traveled 23–160% more distance compared to normobilirubinemic littermates (Stanford et al., 2015). However, a lack of measurements on the energy expenditure in Gunn rats precludes the ability to determine the cause of reduced bodyweight in these animals.

To explore the possibility of greater BMR we investigated mitochondrial function in several tissues. UCB has traditionally been described as toxic to mitochondria because it uncouples oxidative phosphorylation and induces membrane permeabilisation (Noir et al., 1972; Zucker et al., 1992; Rodrigues et al., 2000, Rodrigues et al., 2002b; Jiang and Wang, 2004). For instance, 20–40 µM of UCB reduced state 3 (submaximal oxidative phosphorylation) respiration by 50% with a corresponding increase in the rate of LEAK respiration (uncoupled mitochondrial respiration) via CI and CII in isolated rat mitochondria (Noir et al., 1972). In the present study, exogenous UCB addition to control liver tissue induced dose-dependent inhibition of ETS, and inhibited OXPHOS at concentrations of 125 µM UCB (see Figure 1A) which is above the reference range and potentially toxic in humans (Jansen, 1999). Furthermore, 125 µM UCB affected the membrane integrity of mitochondria, as demonstrated by a 59% increase in O_2_ flux with cytochrome c addition (Figure 1D) and may indicate that UCB induces apoptosis at this concentration through the mitochondrial pathway. This is consistent with previous studies that show that UCB increases mitochondrial membrane permeability and induces release of cytochrome c/apoptosis (Rodrigues et al., 2000; Rodrigues et al., 2002b; Naveenkumar et al., 2015).

Surprisingly, the rate of LEAK respiration was not affected by UCB, nor was OXPHOS inhibited at concentrations less than 125 µM (Figure 1A). This is contrary to previous studies that demonstrate an uncoupling effect of UCB on respiration and inhibition of oxidative phosphorylation in the presence of 10–100 µM UCB (Mustafa et al., 1969; Noir et al., 1972). This inconsistency may be explained by the concentration of albumin used in experimental models, as albumin avidly binds UCB and limits its diffusion into mitochondria (Vitek and Ostrow, 2009). For example, in the absence of albumin, free UCB induces mitochondrial uncoupling, however, it has no effect on mitochondrial function at 1:1 UCB: albumin molar ratios (Mustafa et al., 1969; Noir et al., 1972). This study used a final albumin concentration of 15 µM (1 g L^−1^) with UCB: albumin molar ratios that are greater than 1, indicating that significant mitochondrial dysfunction was induced above a 8:1 UCB: albumin ratio (125 µM UCB). These molar ratios are higher than that found *in vivo* in the circulation of Gunn rats and would likely lead to greater UCB transfer and accumulation into the tissue (Odell, 1966). Understanding the physiological importance of this finding remains an important research question requiring further investigation of variable tissue UCB concentrations.

The impact of UCB on mitochondrial function has been investigated extensively *in vitro*. However, few studies have evaluated mitochondrial function in hyperbilirubinemic animals, and these investigations report inconsistent results (Fritz-Niggli, 1968; Celier et al., 1992; Zelenka et al., 2016). Celier et al., (1992) showed that isolated hepatic mitochondria from male Gunn and wild type control rats (8 weeks of age) have comparable state 3 O_2_ flux and respiratory control ratios (RCRs). Conversely, Fritz-Niggli (1968) reported reductions in P/O ratios in male and female Gunn rats compared to Sprague–Dawley controls in isolated hepatic mitochondria indicating that Gunn rats have reduced capacity for oxidative phosphorylation. It is important to note that the serum bilirubin concentrations of the Gunn rats from Fritz-Niggli (1968) ranged between 115–240 μM, whilst those within this study approximated 80–100 µM (Table 1). These bilirubin concentrations are more likely to affect mitochondrial function and may contribute to the discrepancies between studies (Rossi et al., 2005). Unlike the other two studies that report no change or a decline in mitochondrial function, Zelenka et al., (2016) demonstrated that O_2_ flux of isolated hepatic mitochondria from aged (12–18 months old) Gunn rats was greater when compared to normobilirubinemic siblings. In the present study we did not report any changes to mitochondrial O_2_ flux (relative to tissue mass or citrate synthase activity) in hepatic tissue or skeletal muscle between adult (∼3.5 months old) hyperbilirubinemic Gunn and normobilirubinemic littermates which is in agreement with Celier et al., (1992). Inconsistent findings between studies could be caused by differences in the degree of hyperbilirubinemia—although this cannot be confirmed because bilirubin concentrations were not reported outside of Fritz-Niggli (1968)—methods employed, and the age of the animals studied. Considering that this is the first study of hyperbilirubinemic rats to measure mitochondrial function in additional tissue (i.e. skeletal muscle) beyond the liver, it gives us confidence that the degree of hyperbilirubinemia reported in this model does not influence mitochondrial O_2_ flux.

Conversely, CI+II OXPHOS relative to ETS (CI+II OXPHOS FCR) was significantly greater in hepatic tissue of female Gunn rats (Figure 2B) and this was also observed when liver tissue of normobilirubinemic female rats was treated with 31 or 62.5 µM exogenous UCB (Figure 1C). O_2_ flux is affected by mitochondrial density and mitochondrial quality (i.e. function per individual mitochondria). Therefore, FCRs such as OXPHOS: ETS ratios, eliminate the influence of mitochondrial density allowing the study of qualitative changes in the electron transfer pathways involved in the electron transfer system (Gnaiger, 2014). Increased CI+II OXPHOS FCR indicates that hepatic mitochondria in female Gunn rats are working closer to their maximal oxidative capacity and may indicate greater mitochondrial energy efficiency (Gnaiger, 2014; Holland et al., 2018). Increased OXPHOS respiration, without a change in ETS, reflects greater coupling of mitochondrial respiration to ATP synthesis (i.e. greater mitochondrial efficiency) and causes a reduction in reserve oxidative capacity (Lemieux et al., 2011; Gnaiger, 2014; Greggio et al., 2017; Holland et al., 2018). Conversely, enzymatic defects in mitochondrial respiratory complexes (CI-IV), independent of ATP synthesis, limits ETS respiration and reduces reserve oxidative capacity (Lemieux et al., 2011; Gnaiger, 2014). Female Gunn rats demonstrated a small non-significant increase in CI+II OXPHOS and a decrease in ETS (Figure 2). Therefore, these results suggest that the reason of increased hepatic CI+II OXPHOS FCR in female Gunn rats was due to a combination of improved mitochondrial efficiency and enzymatic dysfunction.

Although, a similar effect was reported when control liver tissue was treated with exogenous UCB (31 or 62.5 µM), reduction in reserve oxidative capacity in this instance was likely caused by UCB-mediated inhibition of mitochondrial respiratory complexes. Exogenous UCB treatment dose-dependently inhibited ETS causing an increase in CI+II OXPHOS FCR (Figure 1A). Therefore, reduction in ETS respiration suggests that UCB inhibits activity of one or more of the mitochondrial complexes (Lemieux et al., 2011; Gnaiger, 2014). This conclusion is supported by a study showing that UCB inhibits CIV activity by 18–20% in liver tissue at 1:2 UCB: albumin molar ratios (Malik et al., 2010). Since this study employed UCB: albumin molar ratios greater than 1:2, exogenous UCB treatment likely inhibited CIV activity in liver tissue causing a reduction in ETS capacity.

To explore further potential mechanisms that could explain the reduced adiposity in female Gunn rats, we measured hepatic gene expression related to mitochondrial biogenesis. PGC-1α is a co-activator that interacts with transcription factors to modulate gene expression of mitochondrial biogenesis and fatty acid metabolism (Scarpulla, 2011). For instance, PGC-1a induces NRF1 gene expression and interacts with NRF1 protein to induce mitochondrial biogenesis (Wu et al., 1999). *PGC-1α* expression was significantly increased in female Gunn rats with a similar trend (*p* = 0.11) in male Gunn rats compared to controls (Figure 7). Furthermore, we found a near two-fold (non-significant, *p* = 0.09) increase in *NRF1* expression in female Gunn rats which is consistent with elevated *PGC-1α* expression (Figure 7). Therefore, elevated *PGC-1α* and *NRF1* gene expression suggests that there is increased hepatic mitochondrial biogenesis in female Gunn rats (Scarpulla, 2011).

To evaluate the possibility of increased mitochondrial biogenesis in Gunn rats, we measured citrate synthase activity and the protein expression of subunits representative of mitochondrial respiratory complexes in liver tissue. Although there was no change in citrate synthase activity, there was a significant increase in CIV subunit 1 expression in female Gunn rats. Elevated CIV subunit 1 expression is consistent with greater *PGC-1α* and *NRF1* expression indicating that there may be increased hepatic mitochondrial biogenesis in female Gunn rats. Considering that UCB can inhibit CIV activity, greater CIV subunit 1 expression in female Gunn rats could represent a compensatory mechanism to UCB-mediated inhibition (Vaz et al., 2010; Malik et al., 2010). Surprisingly, a similar effect was not observed in male Gunn rats suggesting that changes to CIV subunit 1 expression could be caused by other factors. In addition to UCB, UGT1A1 is an important enzyme for 17β-estradiol conjugation and excretion through the hepatobiliary route (Zhou et al., 2011; Sambasivarao, 2013). Consequently, UGT1A1 impairment in female Gunn rats potentially increases estrogen concentrations, however, this remains to be determined. Galmés-Pascual et al., (2017) demonstrated that 17β-estradiol administration in ovariectomized rats increased hepatic CIV activity and protein expression. Furthermore, incubating HepG2 cells with 17β-estradiol increases CIV expression and ATP levels compared to non-treated cells (Galmés-Pascual et al., 2017). Although pAMPK:AMPK ratios were not different, CIV subunit 1 expression was increased in female Gunn rats, indicating that 17β-estradiol levels may be elevated in this animal model, and requires assessment in future studies.

## Conclusion

Hyperbilirubinemic Gunn rats displayed a strong sexually dimorphic effect on body composition, fatty acid metabolism, and hepatic mitochondrial biogenesis. While all Gunn rats demonstrated reduced bodyweight and lean mass compared to normobilirubinemic controls, only females had reduced fat mass and hepatic triglyceride concentrations with a trend toward increased β-oxidation gene expression. Reduced fat mass could potentially be explained by elevated hepatic β-oxidation, reduced food intake, differences in lean mass constitution, or increase in hepatic mitochondrial biogenesis. Female Gunn rats demonstrated increased hepatic CI+II OXPHOS FCR and this was associated with elevated *PGC-1*α and greater CIV subunit 1 expression which could have developed as an adaptive response to UCB-mediated inhibition. The absence of significant effects in male Gunn rats suggests an interaction with reproductive hormones including estrogen and warrants further investigation.

## Data Availability

The raw data supporting the conclusion of this article will be made available by the authors, without undue reservation, to any qualified researcher.
