# Supplementary material for: Mitochondrial Function, Fatty Acid Metabolism, and Body Composition in the Hyperbilirubinemic Gunn Rat
Source: Front Pharmacol. 2021 Mar 8;12:586715. doi: 10.3389/fphar.2021.586715 (PMC7982585; doi:10.3389/fphar.2021.586715)
Supplement: Supplementary file 1 [file datasheet1.pdf]

Supplementary Material

1 Methods

1.1 Additional procedures:

Animals were gavaged daily with 400μL of drinking water and sterile phosphate buffered saline (Gibco®, United Kingdom) was administered every 2 days via an intraperitoneal injection from day 0 to day 16. Blood samples (approx. 1 mL) were collected at Day 1 and Day 8, after a 6-7 hour fast, via tail bleed. Animals were fasted for 5-7 hrs prior to blood collection. Animals were then anesthetized with 2-5% isofluorane (Pharmachem, Australia) in 100% oxygen via inhalation (1-2 L min^{-1}). Pedal reflexes were tested to ensure loss of pain responses. Blood was drawn via gentle massage of the tail after removal of the tail tip (1-2 mm). The first drop of blood was discarded before collecting 1 mL of blood in a 1.5 mL microtube. On the final day animals were euthanized via 50 mg kg^{-1} pentobarbitone injection (Pharmachem) and removal of the heart.

Table S1. List of Primers for qPCR.

Gene	Forward	Reverse
ACOX1	AGTCTGAAATCAAGCAAAGC	CATTAATTCGAAGGTAGGTCTC
ACADVL	CAAGATCTGGATCAGTAATGG	AAGCTGTGATCTTCTCTTTC
B2M	ACTGGTCTTTCTACATCCTG	AGATGATTCAGAGCTCCATAG
CPT1a	CACTGATGAAGGAAGAAGAC	CCAGTCACTCACGTAATTTG
FASN	AAAAGGAAAGTAGAGTGTGC	GACACATTCTGTTCACTACAG
FGF21	CAAATCCTGGGTGTCAAAG	AAAGTGAGGCGATCCATAG
HADHA	ACAGGTTTACAAAACAGTGG	CTCTCCAAATTTCTCTGATTCG
NRF1	TTATCCCAGAGATGCTCAAG	CATGAAACCCTTTGCTTTTG
PGC-1α	AGGTATGACAGCTATGAAGC	GTGTCAGGTCTGATTTTACC

Note: ACADVL, acyl-CoA dehydrogenase, very long chain; ACOX1, acyl-CoA oxidase 1; B2M, β-2-microglobulin; CPT1a, carnitine palmitoyltransferase 1A; FASN, fatty acid synthase; FGF21, fibroblast growth factor 21; HADHA, hydroxyacyl-CoA dehydrogenase trifunctional multienzyme

complex subunit alpha; NRF1, nuclear respiratory factor 1; PGC-1α, peroxisome proliferative activated receptor gamma coactivator 1 alpha.

2 Results:

Table S2. Offspring distribution of homozygote and heterozygote Gunn rats.

**Variable**	**Phenotype**		**n**	**χ^2 (df)**	**P value†**
	Het n [%]	Hom n [%]			
Offspring					
• Males	15 [21]	20 [28]	72	2.11 (3)	0.55
• Females	22 [31]	15 [21]			

Note: Het, heterozygote (normobilirubinemic) Gunn rats; Hom, homozygote (hyperbilirubinemic) Gunn rats. †Chi-square test for independence.

Table S3. Organ weights relative to bodyweight of hyperbilirubinemic and normobilirubinemic rats.

Variable	Phenotype		P value
	Control (n=18)	Gunn (n=19)	
**Liver/Bodyweight (mg g^{-1})**			
• Males	34.4 (1.98)	36.4 (2.81)	0.16
• Females	34.2 (2.99)	46.6 (3.23)	**<0.001**
**Heart/Bodyweight (mg g^{-1})**			
• Males	2.77 (0.20)	2.84 (0.23)	0.58
• Females	3.18 (0.34)	3.59 (0.35)	**<0.05**
**Lungs/Bodyweight (mg g^{-1})**			
• Males	3.60 (0.24)	3.64 (0.30)	0.8
• Females	4.46 (0.50)	5.13 (0.52)	**<0.01**
**Kidney/Bodyweight (mg g^{-1})**			
• Males	3.78 (0.24)	4.06 (0.42)	0.17
• Females	3.80 (0.40)	4.13 (0.32)	**<0.05**
**Spleen/Bodyweight (mg g^{-1})**			
• Males	2.71 (0.38)	2.74 (0.26)	0.88
• Females	3.11 (0.41)	3.90 (0.61)	**<0.01**
**Testes/Bodyweight (mg g^{-1})**			
• Males	4.48 (0.39)	4.75 (0.57)	0.49
**Soleus/Bodyweight (mg g^{-1})**			
• Males	0.40 (0.03)	0.35 (0.03)	**<0.01**
• Females	0.40 (0.04)	0.41 (0.06)	0.74
**EDL/Bodyweight (mg g^{-1})**			
• Males	0.34 (0.03)	0.32 (0.04)	0.42
• Females	0.35 (0.03)	0.34 (0.04)	0.66

Note: Control group represents normobilirubinemic heterozygote littermates. Gunn group represents hyperbilirubinemic homozygote littermates. Values are represented as mean (standard deviation). All comparisons are made between phenotype within the same sex.

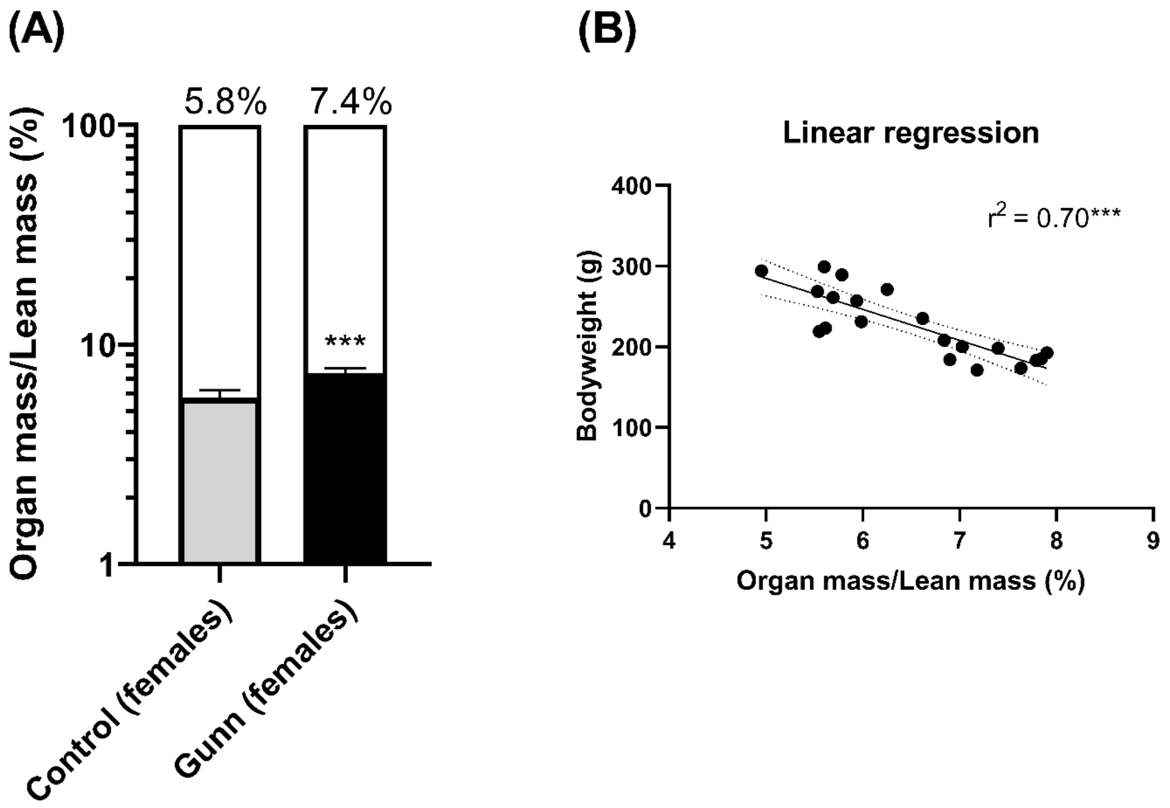

Figure S1. The relative proportion of organ mass to total lean mass in female hyperbilirubinemic (Gunn) and normobilirubinemic (control) rats (A) and the relationship of this ratio to bodyweight (B). Note: total organ mass was a sum of liver, lungs, heart, kidneys, and spleen mass. Data are presented as mean (standard deviation). P<0.05*, <0.01**, <0.001*** compared to control.
